# Aortic arch thrombosis complicated by an embolic stroke in a patient with COVID-19: A case report

**DOI:** 10.1016/j.amsu.2021.102760

**Published:** 2021-08-23

**Authors:** Abdulrahman F. Al-Mashdali, Husam N. Al-Dubai, Akram F. Al-Warqi

**Affiliations:** a Internal Medicine Department, Hamad Medical Corporation, Doha, Qatar; b Clinical Radiology Department, Hamad Medical Corporation, Doha, Qatar

**Keywords:** COVID-19, Aortic thrombosis, Stroke, Coagulopathy, Case report

## Abstract

**Introduction:**

Aortic thrombosis is an uncommon condition with serious embolic complications. COVID-19 is currently recognized to be associated with both venous and arterial thrombosis. However, only a limited number of COVID-19 cases associated with aortic thrombosis have been reported in the literature since the beginning of the pandemic.

**Case presentation:**

A 66-year-old lady was admitted to our hospital with acute ischemic stroke. Floating aortic arch thrombus was detected incidentally on CT imaging. Interestingly, the patient reported a history of fever and cough and was found to have COVID-19 pneumonia based on nasopharyngeal polymerase chain reaction (PCR) and imaging. The patient received three months of anticoagulant therapy, and repeated imaging study did not reveal any aortic thrombus.

**Clinical discussion:**

COVID-19 related aortic thrombosis has been reported chiefly in severe cases. The SARS-CoV-2 can directly infect the endothelium of the vessels, which might explain the occurrence of arterial thrombosis in milder COVID-19 cases with the absence of the hyperinflammatory state. The management guideline for aortic thrombosis is scarce and based only on case reports and series.

**Conclusion:**

Aortic thrombosis is a devastating condition that can be easily missed without clinical suspicion. Our patient developed acute ischemic stroke, most likely embolic originating from the aortic thrombus. The clinician should consider this condition in any COVID-19 patient presenting with thromboembolic events, such as stroke or acute limb ischemia. Further study is required to explain the pathophysiology of arterial/venous thrombosis in mild-moderate COVID-19 cases.

## Highlights

- Aortic thrombosis is a rare condition with serious embolic complications.
- COVID-19 is an emerging condition associated with both venous and arterial thrombo-embolic events.
- A limited number of COVID-19 cases associated with aortic thrombosis have been reported in the literature.
- Computed tomography (CT) of aorta should be considered in the evaluation of stroke in COVID-19 patients to rule out aortic thrombo-embolism.
- The management guidelines of aortic thrombosis are controversial, and based mainly on case reports and series

## 1 Introduction

Since the end of 2019, severe acute respiratory syndrome coronavirus 2 (SARS-CoV-2) infection has spread worldwide, and more than 180 million confirmed cases of coronavirus disease 2019 (COVID-19) have been reported globally. The clinical spectrum of SARS-CoV-2 infection ranges from asymptomatic infection to critical and fatal illness. Although COVID-19 primarily affects the respiratory system, patients may suffer from various complications that involve different organ systems. Hypercoagulability is a well-recognized complication of COVID-19, resulting in either arterial or venous thromboembolic complications [1].

Aortic thrombosis is a rare condition that can lead to catastrophic systemic embolization. Typically, aortic thrombosis is related to hypercoagulable states. However, it can be associated with malignancy and different aortic disorders (such as aortic atherosclerosis, aortic dissection, or aortic aneurysm). Since the beginning of the current COVID-19 pandemic, aortic thrombosis has been increasingly reported in the literature, particularly with severe COVID-19 cases [[2], [3], [4]].

Herein, we report an elderly woman who was admitted to our hospital with a diagnosis of possible ischemic stroke. A floating aortic arch thrombus was detected incidentally by CT imaging of cranial arteries. The patient reported a history of fever and cough for few days before this presentation and tested positive for COVID-19 with the typical radiological features of COVID-19 pneumonia. This case report has been reported in line with the SCARE Criteria [5].

## 2 Presentation of case

A 66-year-old lady, with a past medical history of hypertension and type 2 diabetes mellitus, presented to the emergency department (ED) with slurred speech and right-sided weakness that had started abruptly 2 h before the presentation while she was sitting on the chair. There was no history of headache, convulsion, or loss of consciousness. However, the patient reported a history of fever and cough over the last few days before this presentation, but she did not seek medical advice for that. She denied smoking or alcohol intake. Also, she denied personal or family history of stroke, thromboembolic event, or cardiovascular diseases. In ED, the patient was vitally stable (temperature of 36.8 °C, blood pressure of 135/78 mmHg, pulse rate of 82 beats/minute, respiratory rate of 16 cycles/minute, oxygen saturation of 96% on room air). Neurological examination was significant for dysarthric speech, right upper motor neuron facial palsy, and dense right-sided hemiplegia (power was 0–1/5 in both right upper and lower limbs based Medical Research Council grade for muscle strength). Physical examination of other body systems was unremarkable.

**Fig. 1:**
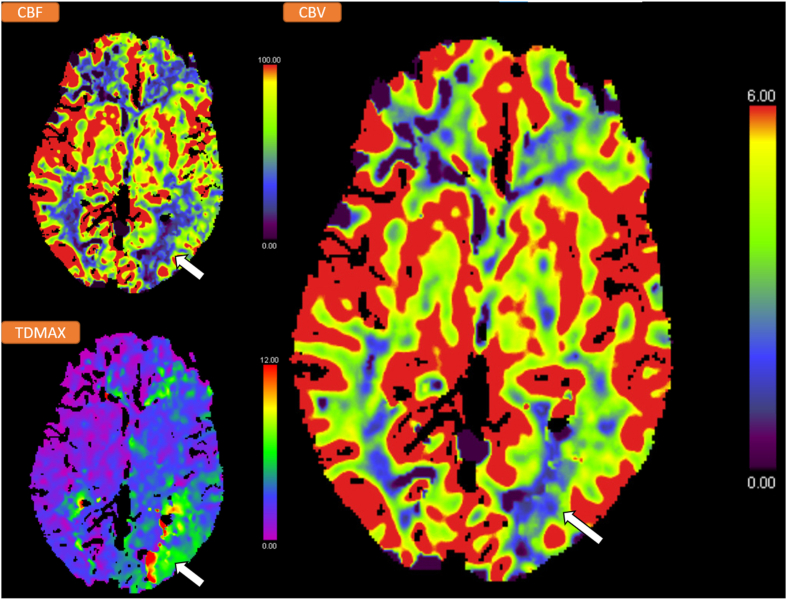
Axial computed tomography (CT) scan of the brain with perfusion images revealing left parieto-occipital increase in the TDMAX and CBF with decrease in the CBV (white arrows) suggestive of small core infarction with surrounding penumbra (brain tissues at risk). CBF: Cerebral blood flow; CBV: Cerebral blood volume; TDMAX: Time to maximum.

**Fig. 2:**
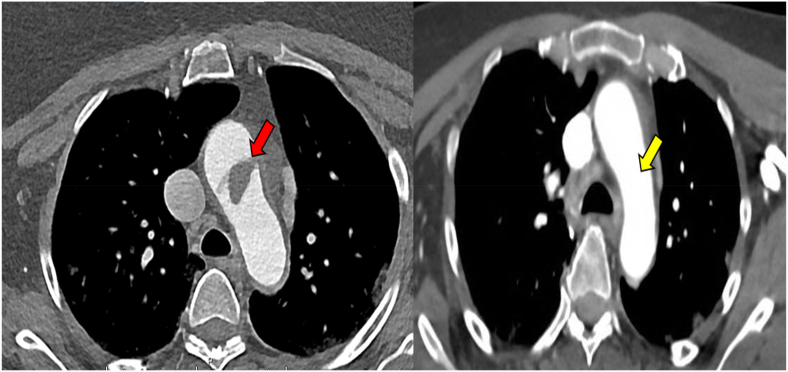
Axial CT scan of the chest with contrast in soft tissue/bone window that shows an aortic arch partial filling defect (red arrow) suggestive of thrombus formation with resolution of the thrombus 10 days later (yellow arrow) after anticoagulant therapy. (For interpretation of the references to colour in this figure legend, the reader is referred to the Web version of this article.)

**Fig. 3:**
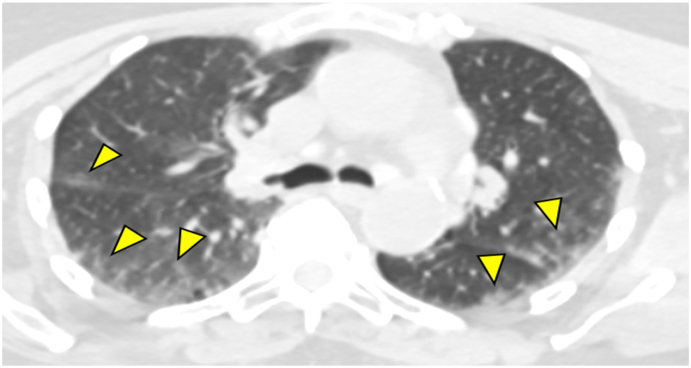
Axial CT of the chest (lung window) shows multiple bilateral subpleural and peripheral ground glass opacifications and atelectatic changes (yellow arrow heads) suggestive of COVID-19 pneumonia. (For interpretation of the references to colour in this figure legend, the reader is referred to the Web version of this article.)

Brain CT (Computed Tomography) with CT angiography (with contrast) of cerebral arteries was done urgently and revealed evidence of acute ischemic stroke in the left middle cerebral artery territory (Fig. 1). Incidentally, the lower cuts of CT angiography of cerebral arteries depicted a large floating thrombus in the proximal aortic arch (Fig. 2). Furthermore, bilateral pulmonary peripheral ground-glass opacifications were observed suggestive for COVID-19 pneumonia (Fig. 3). Initial laboratory investigations revealed normal complete blood count, coagulation profile, and comprehensive metabolic panel. Further blood tests showed a slight increase in C-reactive protein, D-dimer, Interleukin-6, and LDH, but serum ferritin level was within the normal range (Table 1). COVID-19 PCR (polymerase chain reaction) test was positive. Electrocardiography (ECG)and echocardiography were unremarkable with no evidence of the cardioembolic source.

**Table 1:** Laboratory findings on admission.

Parameter	Value	Reference Range
WBC	5.7 × 10^3/uL	4.0–10.0 × 10^3/uL
Hgb	12.3 gm/dL	13.0–17.0 gm/dL
Platelet	401 × 10^3/uL	150–400 × 10^3/uL
PT	11.3 seconds	9–12 seconds
PTT	24.9 seconds	24–32 seconds
INR	1	0.8–1.2
D-Dimer	0.68 mg/L	0.00–0.46 mg/L
Fibrinogen	3.14 gm/L	1.70–4.20 gm/L
Urea	2.8 mmol/L	2.5–7.8 mmol/L
Creatinine	66 μmol/L	62-106 μmol/L
ALT	9 U/L	0–41 U/L
AST	14 U/L	0–40 U/L
CRP	20.1 mg/L	0.0–5.0 mg/L
LDH	252 U/L	135–214 U/L
Ferritin	303 μg/L	18-340 μg/L
Interleukin-6	16 pg/mL	≤7 pg/mL

WBC: White blood cell; Hgb: Hemoglobin; INR: International normalization ratio; PT:Prothrombin time; APTT: activated partial thromboplastin time; ALT: alanine aminotransferase; AST: aspartate aminotransferase; CRP:C-reactive protein; LDH: lactate dehydrogenase.

Accordingly, the patient was admitted to our stroke unit, and therapeutic enoxaparin (1 mg/kg every 12 hours) was started. The vascular surgery team recommended to continue anticoagulation for three months with no need for surgical intervention. The patient was transferred to the COVID-19 facility; however, because she was asymptomatic, she did not receive any regimen for COVID-19. Seven days later, enoxaparin was stopped, and the patient was commenced on Rivaroxaban. A repeated CT aortogram confirmed aortic arch mural thrombus resolution on day 10 of hospital stay (after starting anticoagulation). The patient's condition was discussed again with the neurology and vascular surgery teams, and the final decision was to continue anticoagulation for three months.

After two weeks of isolation, the repeated COVID-19 PCR test was negative, and isolation was discontinued. The patient was transferred to our rehabilitation Institute for active inpatient physiotherapy and rehabilitation program. Extensive screening for hypercoagulable disorders (including antiphospholipid syndrome, hyperhomocysteinemia, Factor V Leiden, and protein C and S activities) was performed, and all were negative (Of note, blood samples were extracted before starting the anticoagulant; however, the results came back few weeks after that). CT aortogram was repeated after three months of anticoagulant therapy and did not show any evidence of aortic thrombosis. Currently, the patient is doing well with minimal residual right-sided weakness requiring some physical support. She was kept on antiplatelet therapy according to the stroke team recommendation.

Patient perspective: “I heard before about lower limbs venous clot during pregnancy, but I was not aware about the possibility arterial clotting and its dangerous complications. I realized that COVID-19 is a dangerous disease which can lead to variable health problems, like my condition."

## 3 Discussion

Aortic thrombosis is an uncommonly encountered condition during medical practice, and the exact incidence is still unknown. The aorta is characterized by its high blood follow and pressure, making the formation of mural thrombus relatively implausible [6]. In the majority of cases, the aortic thrombus was detected incidentally through workup imaging for embolic complications [3,4,6,7]. Hypercoagulable states are the most common predisposing factors for arterial thrombosis. Certain thrombophilia, particularly antiphospholipid antibody syndrome (APS) and hyperhomocysteinemia, have a definite role in the pathophysiology of arterial thrombosis [8,9]. On the other hand, the role of other thrombophilia in developing arterial thromboembolism, such as protein C or S deficiency and factor V Leiden deficiency, is less clear [10]. In addition, aortic diseases (such as aortic dissection, aortic aneurysm, or aortic atherosclerosis) can predispose to aortic thrombus formation [[10], [11], [12]]. The most devastating complication of aortic thrombosis is distant embolization to different parts of the body that can lead on most occasions to ischemic stroke, acute limb ischemia, or mesenteric ischemia. It was estimated that more than 70% of aortic thrombosis cases develop embolic complications [13].

COVID-19 is a hypercoagulable condition frequently associated with venous thrombotic events, most commonly deep vein thrombosis and pulmonary embolism. Thrombotic events are estimated to occur in around 30% of patients with COVID-19, predominantly in severe cases. Though less common, arterial thrombotic events have also been reported in COVID-19 and mainly involved the arterial supplies of the brain and extremities [2,14]. However, aortic thrombosis cases have been rarely described during the COVID-19 era. Several common risk factors can predispose the COVID-19 patient to thrombotic events, including diabetes mellitus, hypertension, cardiovascular diseases, and chronic kidney disease [2,6].

Interestingly, thromboembolic complications have also been reported in severe COVID-19 cases despite being on a therapeutic dose of anticoagulants. The mechanisms of venous thrombosis in COVID-19 are complex, including the three components of the Virchow triad (hypercoagulability, vessel wall damage, and blood stasis). First, hypercoagulability in severe COVID-19 cases is mainly mediated by the hyperinflammatory response that increases clotting factors and activates platelets. In addition, the virus can directly infect the endothelial cells of blood vessels leading to diffuse endotheliitis, which enhances thrombus formation by damaging the vessel wall. Moreover, Immobility during hospital admission can increase the risk of blood stasis and subsequent thrombus formation. However, the pathophysiology of arterial thrombosis in COVID-19 is not well-established but might be theoretically explained by the mechanisms mentioned above [1,6,14].

Given that the sudden onset of limbs weakness, the presence of the aortic thrombus, and the lack of any evidence of intra/extracranial vessel abnormality, we think that our patient developed embolic stroke originated from the floating aortic arch thrombus. The risk factors for aortic thrombosis in our patient, other than COVID-19, are diabetes mellitus, hypertension, and the presence of diffuse atherosclerosis of the aortic arch [2]. Thrombophilia work up came negative for prothrombotic disorders that might predispose to arterial thrombosis. As mentioned before, aortic thrombosis is infrequent compared with cerebral, coronary, or peripheral arterial thromboses. Therefore, the evidence for the management of aortic thrombosis is limited and mainly based on the available case reports and series. Therefore, our vascular surgery team preferred to give a chance to medical treatment, which proved later to be successful. We believe that our case is highly relevant from different aspects. First, our patient had a mild-moderate COVID-19, whereas in most reported cases, acute aortic thrombosis developed in severe COVID-19 cases. In addition, our patient initially presented to the ED with stroke, and the giant floating aortic was incidentally detected (by lower cuts of CT Angio of the cerebral arteries), which could be easily missed without careful observation. Lastly, the conservative medical approach of our case, without the need for surgical intervention, demonstrated to be effective, supporting the growing evidence for medical treatment of such cases.

## 4 Conclusion and learning points

COVID-19 is a hypercoagulable state associated with both arterial and venous thromboembolic events. Most of those events occur in severe COVID-19 cases, although they can occur in milder cases. Aortic thrombosis is a rare but dangerous condition leading to devastating embolic complications. Few cases of acute aortic thromboses have been reported in COVID-19 patients. CT aortogram is the gold standard to detect aortic thrombosis. Clinicians should consider aortic thrombosis in any COVID-19 patient presenting with acute stroke, and a CT aortogram should be a part of stroke evaluation in such patients. Further study is needed to explain the pathophysiology of hypercoagulability in mild-moderate COVID-19 cases.

## Consent for publication

Written informed consent was obtained from the patient for publication of this case report and the accompanying image. A copy of the written consent is available for review by the Editor-in-Chief upon request.

## Ethical approval

Publication of this case was approved by Hamad medical corporation medical research center.

## Availability of data and materials

The datasets used and/or analyzed during the current study are available from the corresponding author on reasonable request.

## Funding statement

Open access funding of this publication was provided by Qatar National Library (QNL).

## Author contribution

AFA involved in patient care, collected data, performed a literature review and wrote the original draft of the manuscript. HNA performed literature and contributed to manuscript writing. AFW provided the radiological images and wrote the figures' captions. All authors reviewed and approved the final version for submission.

## Research registration

Non-applicable.

## Provenance and peer review

Not commissioned, externally peer reviewed.

## Guarantor

Abdulrahman F. Al-Mashdali

## Declaration of competing interest

The authors have no competing of interest to declare.
